# Platform-mediated patient access to apical surgery information on Chinese short-video platforms: a cross-sectional study of clinical accuracy, transparency, and misinformation risk

**DOI:** 10.3389/froh.2026.1870413

**Published:** 2026-06-29

**Authors:** Wang Ting, Duan Hongyu, Zhou Guokai, Ye Mao

**Affiliations:** 1 Department of Endodontics, Shanghai Xuhui District Stomatological Hospital, Shanghai, China; 2 Department of Stomatology, People's Hospital of Xiangyun Affiliated to Dali University, Yunnan, China

**Keywords:** apical surgery, clinical accuracy, misinformation, oral health communication, patient education, short-video platforms

## Abstract

**Introduction:**

Short-video platforms are increasingly utilized as preliminary sources of oral health information, yet the clinical value of patient-facing content varies across platforms. This study evaluated and compared the educational usability, clinical accuracy, transparency, and misinformation risk of apical surgery videos across four major Chinese social media platforms.

**Methods:**

A standardized search using the term “根尖手术” (apical surgery) was conducted on March 15, 2026, across TikTok, Bilibili, Kwai, and WeChat Channels. To minimize algorithmic bias, newly registered accounts under default sorting criteria were used to select 150 consecutive eligible videos per platform (*N* = 600). Videos were evaluated using the Patient Education Materials Assessment Tool (PEMAT), a study-specific 12-item clinical accuracy checklist, and JAMA benchmarks. Multivariable linear regression identified independent predictors of clinical accuracy.

**Results:**

Bilibili and WeChat Channels demonstrated significantly higher educational usability and clinical accuracy than TikTok and Kwai (*P* < 0.001). Median PEMAT understandability scores were 85% for Bilibili and 82% for WeChat Channels, versus 68% for TikTok and 70% for Kwai. Mean clinical accuracy scores (out of 24) were 20.1 ± 2.8 and 19.5 ± 3.0 for Bilibili and WeChat Channels, compared to 12.4 ± 3.2 for TikTok and 12.6 ± 3.4 for Kwai. Sourcing transparency was generally deficient; only 9.2% of the total sample provided references to evidence sources. Severe misinformation was significantly more prevalent on Kwai (18.7%) and TikTok (15.3%) than on Bilibili (4.0%) and WeChat Channels (5.3%) (*P* < 0.001). Multivariable analysis confirmed that professional uploader status (β = 3.85, *P* < 0.001) and longer video duration (β = 1.72 per 60 s, *P* < 0.001) were independent predictors of superior clinical accuracy.

**Conclusion:**

Patient-directed information regarding apical surgery is highly heterogeneous and strongly modulated by platform ecology. Public health initiatives should encourage qualified endodontic professionals to actively participate in video creation and advocate for platform environments that privilege balanced, source-verified clinical communication.

## Introduction

In recent years, short-video platforms have become an increasingly common source of health information for the public (1–3). In dentistry, many patients use these platforms to search for explanations of unfamiliar procedures, expected outcomes, and postoperative experiences before or after seeing a clinician. Compared with traditional webpages or written patient materials, short videos are easier to access and often more appealing to non-professional audiences. As a result, they now play an important role in shaping how patients first understand many dental treatments.

However, the information patients encounter on these platforms is not presented in a uniform way. What appears first is usually determined by each platform's own recommendation and ranking system, together with its preferred content style and user engagement patterns. In practice, this means that the information available to patients may differ substantially across platforms, even when the same search term is used, driven by distinct algorithmic architectures and user engagement mechanics (4, 5). For medical topics that require balanced explanation of indications, risks, alternatives, and limitations, such variation may influence how well patients understand a procedure and how accurately they form expectations before treatment.

This issue may be particularly relevant in oral healthcare, where many procedures are technically specific and difficult for patients to interpret without professional guidance. Among them, apical surgery is a useful example. Although it is a well-established treatment in endodontic practice, most patients are unfamiliar with it until the procedure is recommended during clinical care. Once they encounter the term, many seek additional information online, often through short-video platforms. The quality of that early information may affect their understanding of the purpose of surgery, its complexity, likely benefits, and possible postoperative sequelae.

Previous studies have shown that online dental information often varies in quality, completeness, and reliability (6–8). Most of this work, however, has focused on websites or general social media content, while evidence on short-video platforms remains relatively limited (9, 10). This is particularly true for apical surgery, here patient-oriented information is often simplified, fragmented, or shaped by the communication style favored by a given platform (11, 12). At present, there is still insufficient evidence on whether different short-video platforms present patients with similarly reliable information, or whether the information environment itself may influence their opportunity to access objective and trustworthy medical content. To address this question, the present study evaluated patient-facing videos on apical surgery across four major Chinese short-video platforms: Chinese TikTok (douyin), Bilibili, Kwai, and WeChat Channels (13–15). By performing a comprehensive cross-platform assessment of educational usability, clinical accuracy, transparency, and misinformation risk, we aimed to examine whether systematic differences exist in the information presented to users across platforms, and how platform-mediated environments influence public access to objective endodontic content.

## Materials and methods

### Study design and video retrieval

This cross-sectional study evaluated patient-facing videos on apical surgery across four major Chinese short-video platforms: Chinese TikTok (Douyin), Bilibili, Kwai, and WeChat Channels. These platforms were selected because they represent the definitive pillars of contemporary Chinese social media, a status heavily documented by the China Internet Network Information Center (CNNIC) regarding their leading market share and role in public digital health communication (16).

A standardized search was conducted on March 15, 2026, using the Chinese term “根尖手术 (apical surgery)”. To minimize the influence of prior browsing history and personalized recommendations, all searches were performed using newly registered accounts with no previous viewing or interaction records. Browser cache and cookies were cleared before each search session. This rigorous protocol follows established methodological precedents in infodemiology designed to effectively isolate and neutralize personalized algorithmic recommendation bias (17, 18). Videos were retrieved using each platform's default comprehensive or relevance-based sorting mode, as this approach was considered most reflective of the information routinely presented to users and therefore suitable for cross-platform comparison of patient-facing information environments (19, 20).

### Eligibility criteria and sample selection

Videos were included if they were in Chinese and provided substantive information related to apical surgery, including indications, procedural description, outcomes, postoperative care, or other content relevant to patient understanding. Videos were excluded if they were duplicates or reposts, shorter than 15 s, unrelated to the topic, lacking meaningful informational content, or purely promotional in nature without educational value. This sampling strategy was intended to approximate the first layer of information most likely to be encountered by routine users under default platform conditions, rather than to characterize the entire archive of platform content (21, 22).

**Figure 1:**
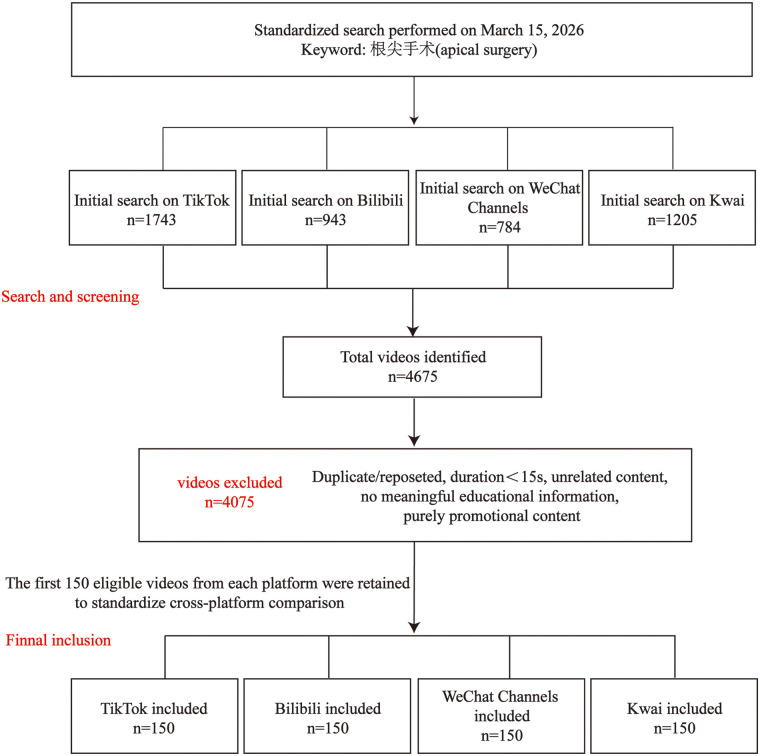
Flowchart of video identification, screening, and inclusion. A standardized search was performed on March 15, 2026, using the Chinese search term “根尖手术” (apical surgery) across Chinese TikTok (抖音), Bilibili (B站), Kwai (快手), and WeChat Channels (微信短视频). A total of 4,675 videos were initially identified. After exclusion of duplicate or reposted videos, videos shorter than 15 seconds, irrelevant content, videos without meaningful educational information, and purely promotional material, 600 videos were included in the final analysis, with 150 videos retained from each platform for cross-platform comparison.

The initial search identified 4,675 videos in total, including 1,743 on TikTok, 943 on Bilibili, 1,205 on Kwai, and 784 on WeChat Channels. After screening according to the predefined criteria, 150 eligible videos were included from each platform, resulting in a final sample of 600 videos. When more than 150 eligible videos were available, the first 150 eligible videos encountered in the standardized search sequence were retained in order to allow balanced comparison across platforms (Figure 1).

### Data extraction

For each included video, the following information was recorded: platform source, duration, upload date, uploader type, and visible engagement indicators such as likes, comments, shares, or equivalent interaction metrics, depending on platform display format. Uploader type was classified as medical professional, academic or institutional account, commercial account, or non-professional/other account. The main content focus of each video was also documented, including whether the video primarily addressed indications, procedural explanation, treatment experience, postoperative issues, or general commentary.

### Video evaluation

Each video was evaluated in four domains: educational usability, clinical accuracy, transparency, and misinformation risk. Educational usability was assessed using the Patient Education Materials Assessment Tool for Audiovisual Materials (PEMAT-A/V), including understandability and actionability (23). Clinical accuracy was assessed with a study-specific 12-item checklist covering key patient-relevant aspects of apical surgery, including indications, procedural explanation, risks, alternatives, prognosis, and postoperative care. Each item was scored from 0 to 2, yielding a total score ranging from 0 to 24, with higher scores indicating better clinical accuracy. Details of the checklist, scoring criteria, and interpretation of total scores are provided in Supplementary Table S1. Transparency was evaluated using the JAMA benchmark criteria, including authorship, attribution, disclosure, and currency (24). Misinformation risk was graded as mild, moderate, or severe according to the extent to which a video contained inaccurate, incomplete, or potentially misleading information that could distort patient understanding of apical surgery (25).

For videos judged to contain misleading or potentially misleading information, the dominant pattern of misleading content was further classified into one or more predefined categories: overstatement of success or prognosis, omission of limitations or contraindications, inadequate discussion of complications or uncertainty, presentation of surgery as broadly applicable or prematurely indicated, promotional framing outweighing educational explanation, and unrealistic postoperative expectations. This supplementary classification was used to characterize how misleading information was expressed across platforms (26).

### Reviewer calibration and reliability

All videos were independently assessed by two reviewers with clinical experience relevant to endodontic practice. Before formal evaluation, 30 videos were used for pilot assessment to refine scoring rules and standardize interpretation of the study instruments. Disagreements were resolved through discussion, and a third reviewer was consulted when necessary. Inter-rater reliability was evaluated using intraclass correlation coefficients for continuous summary scores and Cohen's kappa or weighted kappa for categorical variables, as appropriate. Reliability results for the major study measures are presented in Supplementary Table S2. The same independent review and consensus procedure was applied to the classification of misleading content patterns.

### Statistical analysis

Statistical analysis was performed using SPSS version 26.0 (IBM Corp., Armonk, NY, USA). Continuous variables were presented as mean ± standard deviation or median with interquartile range, depending on data distribution, which was assessed using the Shapiro–Wilk test. Comparisons among the four platforms were performed using one-way analysis of variance or the Kruskal–Wallis test, as appropriate. Categorical variables were compared using the chi-square test or Fisher's exact test.

Because visible engagement metrics differed across platforms, engagement was analyzed as a relative binary variable rather than an absolute count. For each platform, videos were classified as having high or low engagement according to whether the composite engagement indicator displayed on that platform was above or below the platform-specific median of the included sample. For regression analysis, uploader type was further dichotomized into professional and non-professional sources. Professional sources included videos uploaded by medical professionals and academic or institutional accounts, whereas non-professional sources included commercial accounts and non-professional and other accounts. Multivariable linear regression analysis was used to identify factors associated with higher clinical accuracy. A two-sided *P* value < 0.05 was considered statistically significant.

### Ethical considerations

This study used only publicly accessible online videos and did not involve human participants, patient data, or clinical intervention. Therefore, institutional review board approval and informed consent were not required.

## Results

A total of 600 videos were included, with 150 videos analyzed from each platform. The initial search yielded 4,675 records across TikTok, Bilibili, Kwai, and WeChat Channels, and 4,075 were excluded after screening. The study selection process is shown in Figure 1. Most included videos had been uploaded within the previous 18 months (64.2%), without a significant difference among platforms. Video duration, however, differed markedly. Bilibili contained the longest videos, with a median duration of 244.5 s, whereas the corresponding values were 45.0 s for TikTok, 51.5 s for Kwai, and 121.0 s for WeChat Channels (*P* < 0.001). Uploader type and content focus also varied by platform. Bilibili and WeChat Channels included a higher proportion of videos from medical professionals or academic/institutional accounts, and these videos more often focused on indications, procedural explanation, and postoperative care. By contrast, TikTok and Kwai more frequently featured content centered on treatment experience or general commentary (Table 1).

**Table 1:** Platform characteristics of included videos on apical surgery.

Variable	TikTok (*n* = 150)	Bilibili (*n* = 150)	Kwai (*n* = 150)	WeChat Channels (*n* = 150)	*P* value
Videos identified	1,743	943	1,205	784	–
Retention rate (%)	8.6	15.9	12.4	19.1	–
Median duration (s), median (IQR)	45.0 (30.3–74.8)	244.5 (180.8–420.0)	51.5 (35.0–87.8)	121.0 (80.5–183.5)	<0.001
Published within 18 months, *n* (%)	96 (64.0)	97 (64.7)	96 (64.0)	96 (64.0)	0.998
Uploader type, *n* (%)					<0.001
Medical professional	52 (34.7)	86 (57.3)	49 (32.7)	78 (52.0)	
Academic/institutional	11 (7.3)	24 (16.0)	8 (5.3)	21 (14.0)	
Commercial account	46 (30.7)	15 (10.0)	54 (36.0)	19 (12.7)	
Non-professional/other	41 (27.3)	25 (16.7)	39 (26.0)	32 (21.3)	
*Primary content focus, *n* (%)**					<0.001
Indications	38 (25.3)	72 (48.0)	41 (27.3)	66 (44.0)	
Procedure explanation	61 (40.7)	98 (65.3)	59 (39.3)	88 (58.7)	
Treatment experience	72 (48.0)	45 (30.0)	76 (50.7)	51 (34.0)	
Postoperative issues	28 (18.7)	64 (42.7)	31 (20.7)	55 (36.7)	
General commentary	34 (22.7)	29 (19.3)	37 (24.7)	26 (17.3)	

Values are *n* (%) unless otherwise indicated. Categories of content focus were not mutually exclusive. *P* values were calculated using *χ*² test or Kruskal–Wallis test where appropriate.

*, ** Note: Categories of content focus were not mutually exclusive; a single video could be classified under multiple thematic domains, hence percentages within the platform columns may sum to greater than 100%.

**Figure 2:**
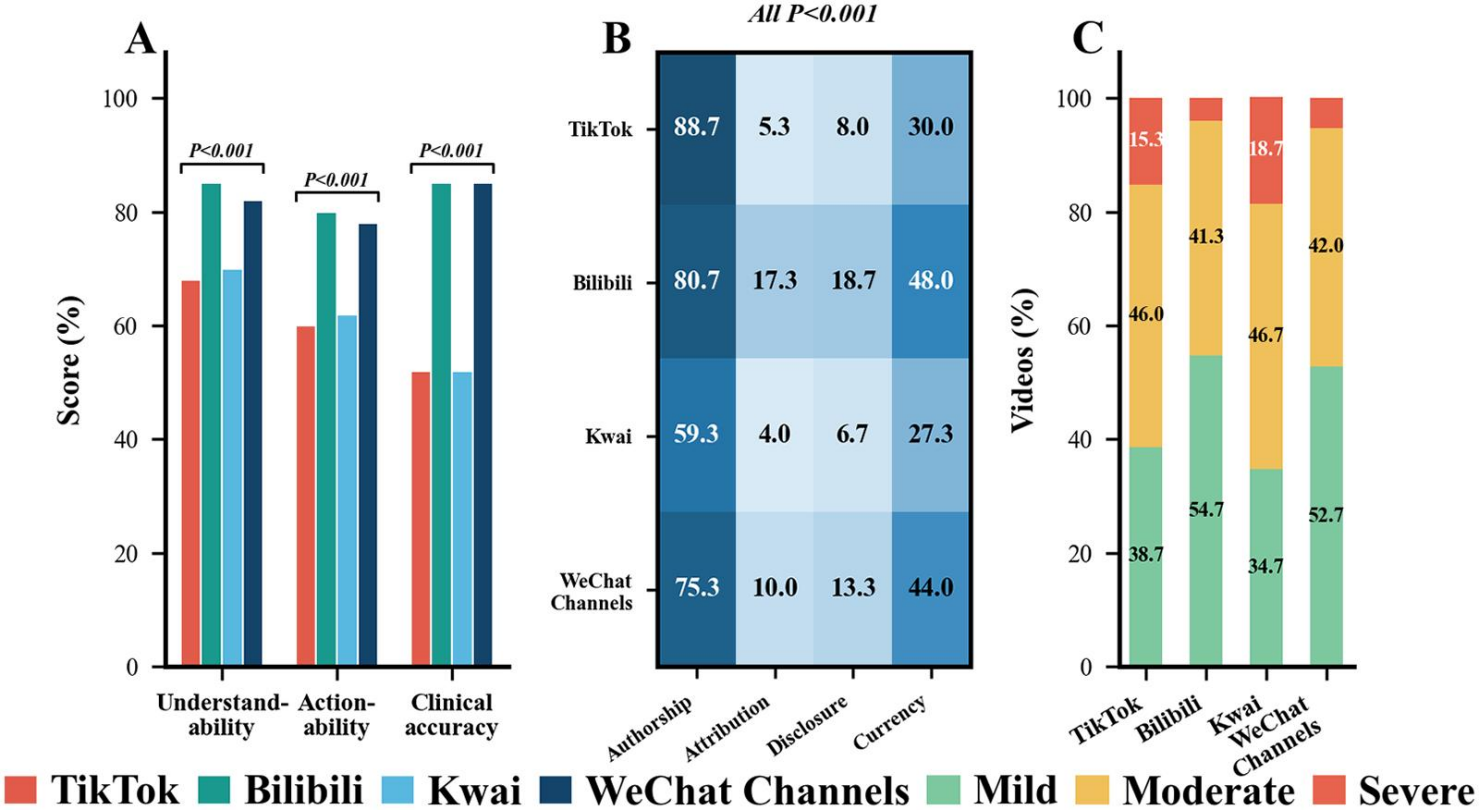
Comparison of educational usability, clinical accuracy, transparency, and misinformation risk across platforms. (**A**) Educational usability and clinical accuracy scores differed significantly across platforms (all *P* < 0.001). (**B**) Transparency indicators based on JAMA criteria, showing proportions of videos reporting authorship, attribution, disclosure, and currency. (**C**) Distribution of misinformation severity (mild, moderate, severe) across platforms. All comparisons were statistically significant (*P* < 0.001).

Substantial between-platform differences were found in educational usability, clinical accuracy, transparency, and misinformation severity (Figure 2; Table 2). PEMAT-A/V scores were higher for Bilibili and WeChat Channels than for TikTok and Kwai. Median understandability scores were 85 for Bilibili and 82 for WeChat Channels, compared with 68 for TikTok and 70 for Kwai (*P* < 0.001). The same overall pattern was observed for actionability. Clinical accuracy followed a similar distribution. Mean scores were 20.1 ± 2.8 on Bilibili and 19.5 ± 3.0 on WeChat Channels, whereas TikTok and Kwai scored 12.4 ± 3.2 and 12.6 ± 3.4, respectively (*P* < 0.001). Transparency indicators were limited across all four platforms. Authorship was commonly identifiable, but attribution to evidence sources was uncommon overall (9.2%). Disclosure and currency were also reported inconsistently, although these items appeared somewhat more often on Bilibili and WeChat Channels. Misinformation severity also differed significantly across platforms. Severe misinformation was most frequent on Kwai (18.7%) and TikTok (15.3%), and less frequent on Bilibili (4.0%) and WeChat Channels (5.3%) (all *P* < 0.001).

**Table 2:** Comparison of educational usability, clinical accuracy, transparency, and misinformation across platforms.

Indicator	TikTok	Bilibili	Kwai	WeChat Channels	*P* value
Educational usability					
Understandability (PEMAT), median (IQR)	68 (60–76)	85 (78–92)	70 (62–78)	82 (74–90)	<0.001
Actionability (PEMAT), median (IQR)	60 (52–70)	80 (72–88)	62 (54–72)	78 (70–86)	<0.001
Clinical accuracy					
Accuracy score (0–24), mean ± SD	12.4 ± 3.2	20.1 ± 2.8	12.6 ± 3.4	19.5 ± 3.0	<0.001
Transparency (JAMA criteria), *n* (%)					
Authorship disclosed	133 (88.7)	121 (80.7)	89 (59.3)	113 (75.3)	<0.001
Attribution provided	8 (5.3)	26 (17.3)	6 (4.0)	15 (10.0)	<0.001
Disclosure stated	12 (8.0)	28 (18.7)	10 (6.7)	20 (13.3)	<0.001
Currency indicated	45 (30.0)	72 (48.0)	41 (27.3)	66 (44.0)	<0.001
Misinformation risk, *n* (%)					<0.001
Mild	58 (38.7)	82 (54.7)	52 (34.7)	79 (52.7)	
Moderate	69 (46.0)	62 (41.3)	70 (46.7)	63 (42.0)	
Severe	23 (15.3)	6 (4.0)	28 (18.7)	8 (5.3)	

Continuous variables compared using Kruskal–Wallis test or ANOVA; categorical variables using *χ*² test.

PEMAT, Patient Education Materials Assessment Tool.

**Figure 3:**
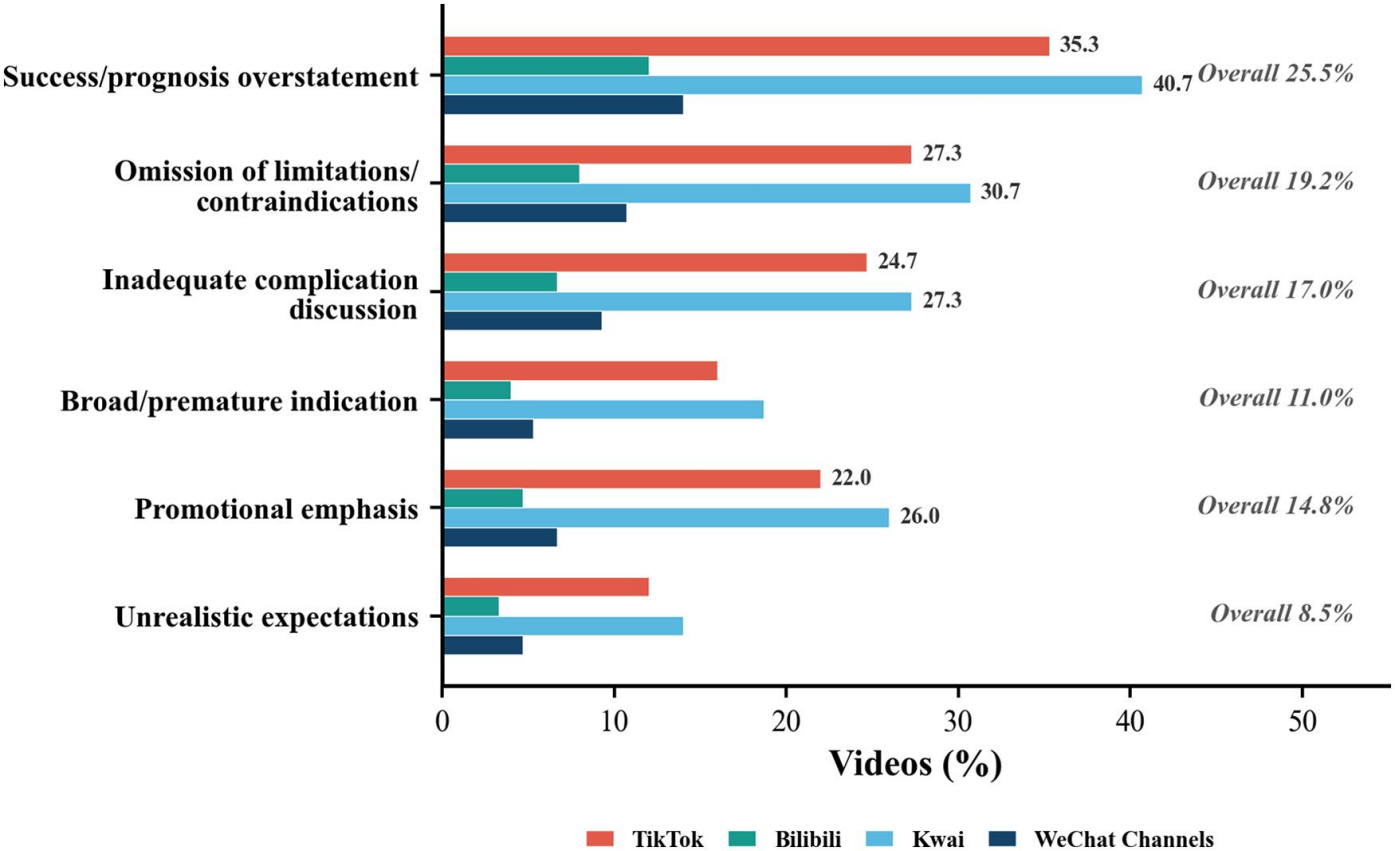
Distribution of misleading content patterns across platforms. The prevalence of six predefined misleading content patterns is shown across platforms. Kwai and TikTok exhibited higher proportions of misinformation, particularly in success/prognosis overstatement and omission of limitations. Overall prevalence rates were highest for success/prognosis overstatement (25.5%) and omission of limitations (19.2%).

Multivariable regression analysis was then performed to examine factors associated with higher clinical accuracy (Figure 3; Table 3). Videos uploaded by medical professionals had significantly higher accuracy scores than those from non-professional sources, with a mean difference of 3.85 points (95% CI, 2.95–4.76; *P* < 0.001). Longer video duration was also independently associated with better accuracy; each additional 60 s corresponded to a 1.72-point increase in score (95% CI, 1.12–2.31; *P* < 0.001). After adjustment, platform remained an independent correlate of accuracy. Compared with TikTok, videos on Bilibili and WeChat Channels showed significantly higher scores (β = 4.12 and 3.75, respectively; both *P* < 0.001), whereas no significant difference was observed between Kwai and TikTok (*P* = 0.698). Engagement was not significantly associated with clinical accuracy (β = 0.28, *P* = 0.392). The overall model was significant and explained a moderate proportion of the variance (adjusted R² = 0.46, *P* < 0.001).

**Table 3:** Multivariable regression analysis of factors associated with higher clinical accuracy.

Variable	β (unstandardized)	Standardized β	95% CI	*P* value
Uploader expertise (professional vs. non-professional)	3.85	0.42	2.95–4.76	<0.001
Video duration (per 60 s increase)	1.72	0.35	1.12–2.31	<0.001
Platform (reference: TikTok)				
Bilibili	4.12	0.38	3.05–5.18	<0.001
Kwai	0.21	0.02	−0.85–1.27	0.698
WeChat Ch`annels	3.75	0.34	2.68–4.82	<0.001
Engagement level (high vs. low)	0.28	0.04	−0.36–0.92	0.392

Model statistics: Adjusted R² = 0.46; overall model *P* < 0.001.

Higher clinical accuracy defined as continuous score (0–24). Linear regression model adjusted for all listed variables.

**Figure 4:**
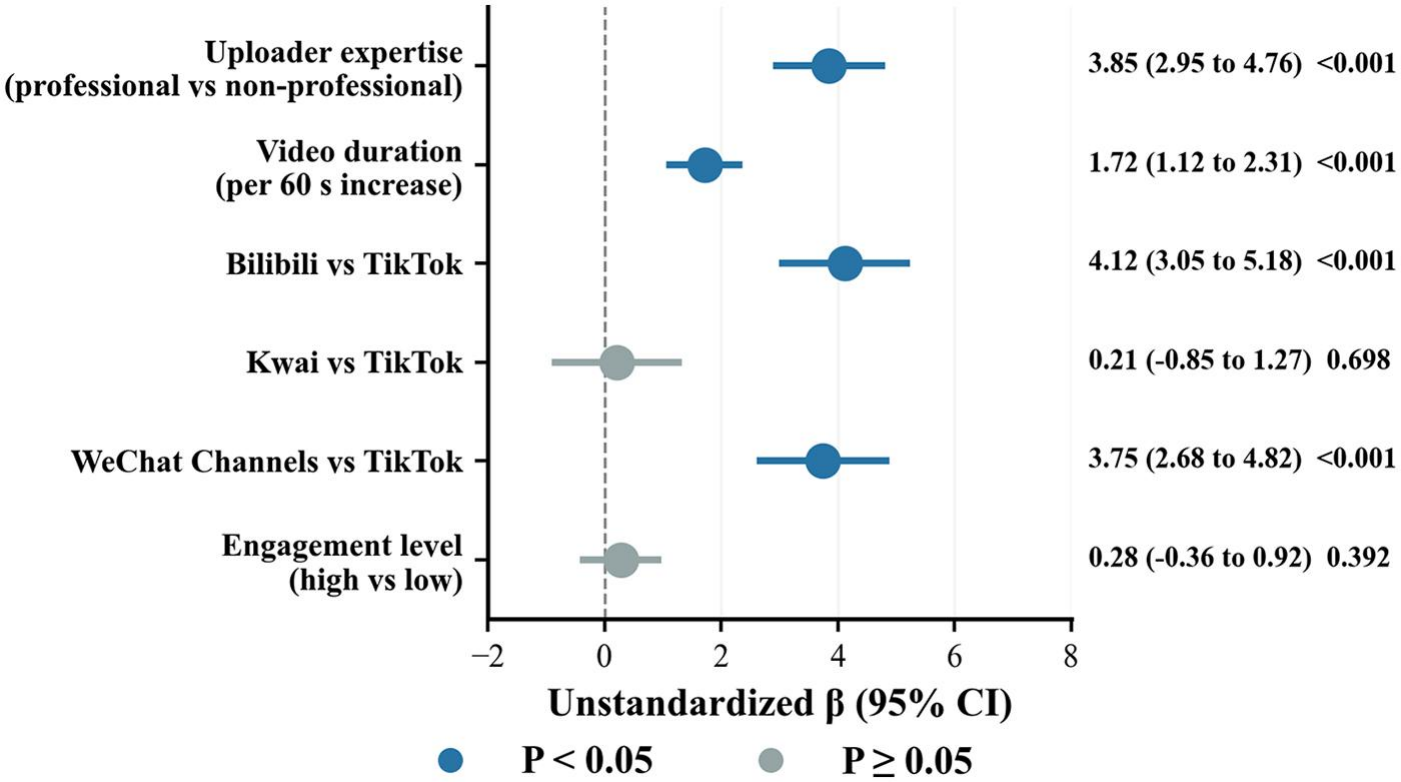
Multivariable regression analysis of factors associated with clinical accuracy. Forest plot showing unstandardized β coefficients with 95% confidence intervals. Professional uploader expertise, longer video duration, and platform (Bilibili and WeChat Channels vs TikTok) were significantly associated with higher clinical accuracy, while engagement level was not statistically significant. Adjusted R² = 0.46; overall model *P* < 0.001.

The pattern of misleading content also varied across platforms (Figure 4; Table 4). Overstatement of treatment success or prognosis was the most frequent problem overall, identified in 25.5% of videos. This pattern was especially common on Kwai (40.7%) and TikTok (35.3%), but less frequent on Bilibili (12.0%) and WeChat Channels (14.0%). Omission of limitations or contraindications was observed in 19.2% of videos overall, and inadequate discussion of complications in 17.0% (27). Both patterns were again more common on TikTok and Kwai. Broad or premature indications were less frequent (11.0%), although the same platform distribution persisted. Promotional framing and unrealistic postoperative expectations were also noted, particularly on TikTok and Kwai. Because these categories were not mutually exclusive, a single video could contribute to more than one type of misleading content.

**Table 4:** Distribution of misleading content patterns across platforms.

Pattern	TikTok (*n* = 150)	Bilibili (*n* = 150)	Kwai (*n* = 150)	WeChat Channels (*n* = 150)	Overall (*N* = 600)
Success/prognosis overstatement	53 (35.3)	18 (12.0)	61 (40.7)	21 (14.0)	153 (25.5)
Omission of limitations/contraindications	41 (27.3)	12 (8.0)	46 (30.7)	16 (10.7)	115 (19.2)
Inadequate complication discussion	37 (24.7)	10 (6.7)	41 (27.3)	14 (9.3)	102 (17.0)
Broad/premature indication	24 (16.0)	6 (4.0)	28 (18.7)	8 (5.3)	66 (11.0)
Promotional emphasis	33 (22.0)	7 (4.7)	39 (26.0)	10 (6.7)	89 (14.8)
Unrealistic expectations	18 (12.0)	5 (3.3)	21 (14.0)	7 (4.7)	51 (8.5)

Categories were not mutually exclusive; one video could contribute to multiple patterns.

## Discussion

This cross-platform study showed that patient-facing videos on apical surgery differed substantially across the four short-video platforms examined. The differences were not confined to style or duration. They were also reflected in educational usability, clinical accuracy, transparency, and the frequency and pattern of misleading content. Overall, Bilibili and WeChat Channels performed better, whereas TikTok and Kwai more often presented shorter and less balanced material. As a result, patients searching for the same topic may encounter very different information environments depending on the platform they use.

One finding worth noting is that misinformation in this setting was usually not expressed as an entirely false statement. More often, it appeared as selective emphasis, omission, or incomplete explanation. Overstatement of treatment success or prognosis was the most common misleading pattern, followed by omission of limitations or contraindications and inadequate discussion of complications. This pattern is clinically relevant. For a procedure such as apical surgery, patient understanding depends not only on whether a statement is technically correct, but also on whether the explanation is sufficiently balanced to support realistic expectations. Content that highlights benefits while leaving out uncertainty, alternatives, or postoperative burden may still distort understanding, even when no single sentence is overtly wrong (28).

The association between uploader type and clinical accuracy is also important. Videos uploaded by medical professionals performed better than those from non-professional sources, and this relationship remained significant after adjustment (29). A similar trend was observed for video duration. Longer videos were more likely to provide accurate and complete information, probably because they allowed more room to explain indications, procedural steps, limitations, and recovery issues. Even so, duration alone was not the main explanation. The effect size for uploader expertise was larger, suggesting that content source may matter more than format length in determining informational quality (30).

Our finding that longer video duration independently correlates with higher clinical accuracy and usability scores invites a valuable contrast with broader global infodemiological literature. For instance, a recent cross-sectional study evaluating YouTube videos on peri-implantitis demonstrated that longer runtimes (between 4 and 20 min) did not necessarily translate to high educational utility, as over half of the videos failed to fall into the “education” category due to the pervasive use of overly technical, inaccessible professional jargon (31). In our sample, however, longer formats on platforms such as Bilibili and WeChat Channels significantly improved both clinical checklist scores and PEMAT understandability. This discrepancy suggests that video length should not be analyzed in isolation from platform ecology; while open global video repositories host highly heterogeneous content types where length may mask raw technical jargon, certain Chinese platforms actively foster a structured “educational tutorial” culture where verified clinicians utilize extended timeframes to systematically deconstruct complex procedures for lay audiences. Furthermore, this cross-study comparison highlights a shifting paradigm in health communication risks. While patients accessing traditional platforms are primarily alienated by inaccessible clinical jargon (31, 32), consumers of short-video platforms like TikTok and Kwai face the inverse hazard: a profound risk of “misleading simplification” characterized by the selective omission of contraindications and the systemic overstatement of prognosis. Consequently, the barrier to oral health equity has evolved from a lack of information or excessive jargon into an algorithmic environment that incentivizes fragmented, attention-driven soundbites over complete, full-spectrum clinical explanations.

These findings also have practical implications for patient protection in digital oral-health communication. For a procedure such as apical surgery, which involves preoperative evaluation, case selection, operative explanation, postoperative care, and follow-up, short-form videos are unlikely to provide a complete educational account. They may serve as an entry point for patient orientation, but they should not be treated as substitutes for comprehensive clinician–patient discussion or structured educational materials. This distinction is especially important because short-video platforms differ from search engines and AI-based conversational systems. Search engines typically return multiple indexed sources that users may compare, and AI chatbots provide synthesized responses whose quality depends on their training and source grounding (33). By contrast, short-video platforms deliver ranked audiovisual content through engagement-sensitive recommendation systems, which may preferentially amplify memorable or emotionally persuasive material even when it is incomplete or weakly sourced. Reducing misinformation on apical surgery will therefore require action at several levels, including stronger verification of professional accounts, greater visibility for videos that disclose sources and limitations, and more active participation by clinicians and professional organizations in producing concise but evidence-based patient-oriented content.

Another point that deserves attention is the weak relationship between engagement and informational value. In this study, videos with higher visible engagement did not show better clinical accuracy. This suggests that popularity on short-video platforms should not be interpreted as a proxy for reliability (34). From a patient perspective, that distinction matters. Highly viewed or frequently liked videos may appear credible at first glance, yet their informational quality may remain limited (35).

The observed platform differences are likely to reflect more than variation in individual uploaders alone. Platform ecology may also play a role. Different platforms favor different content styles, audience expectations, and patterns of circulation. Bilibili and WeChat Channels may provide a comparatively more suitable environment for longer and more explanatory health-related videos, whereas TikTok and Kwai appear to favor brief, experience-centered, and attention-driven formats (36, 37). That difference does not necessarily make one platform inherently reliable and another inherently unreliable, but it may influence how easily balanced medical information can be communicated and encountered (38).

Several limitations should be acknowledged. First, this was a cross-sectional analysis based on videos retrieved at a single time point. Platform content is dynamic, and search results may change over time. Second, only the first 150 eligible videos from each platform were included. This improved comparability, but it may not capture the full range of available content. Third, although structured assessment tools were used, some degree of judgment is unavoidable when evaluating clinical accuracy and misleading framing in patient-oriented material. In addition, we did not formally classify presentation format, such as live procedural footage, animation, slide-based explanation, or board-style teaching. This distinction may influence patient comprehension and should be incorporated in future studies. Nevertheless, the use of predefined criteria and independent review helped improve consistency.

Despite these limitations, the study provides a practical view of the information that ordinary users are likely to encounter when searching for apical surgery on major short-video platforms. The findings suggest that these platforms are not equivalent as sources of patient information. They also indicate that improving online oral-health communication will require more than simply increasing content volume. Greater participation by qualified professionals is important, but so is the broader information environment in which such content is produced, circulated, and prioritized.

## Conclusion

Patient-facing videos on apical surgery differed substantially across the four short-video platforms examined. Bilibili and WeChat Channels generally provided more accurate, usable, and balanced information, whereas TikTok and Kwai more often contained simplified or potentially misleading content. In this setting, misleading information was usually expressed through omission or one-sided emphasis rather than direct factual error. Videos uploaded by medical professionals and videos of longer duration were more likely to achieve higher clinical accuracy. These findings suggest that improving online oral-health communication will require not only greater participation by qualified professionals, but also platform environments that better support clear, complete, and balanced presentation of treatment-related information (39).

## Data Availability

The raw data supporting the conclusions of this article will be made available by the authors, without undue reservation.
