# Supplementary material for: Platform-mediated patient access to apical surgery information on Chinese short-video platforms: a cross-sectional study of clinical accuracy, transparency, and misinformation risk
Source: Front Oral Health. 2026 Jun 29;7:1870413. doi: 10.3389/froh.2026.1870413 (PMC13357420; doi:10.3389/froh.2026.1870413)
Supplement: Supplementary file 1 [file Table1.docx]

**Study-specific clinical accuracy checklist for evaluating patient-facing short videos on apical surgery**

This study-specific checklist was developed to assess the clinical accuracy of patient-facing short videos on apical surgery. The checklist was designed to evaluate whether clinically relevant aspects of apical surgery were presented correctly, sufficiently, and in a manner unlikely to mislead patients. It was used as a topic-specific supplementary tool alongside PEMAT-A/V, JAMA benchmark criteria, and misinformation severity assessment.

| **Item** | **Domain** | **0 points** | **1 point** | **2 points** |
| --- | --- | --- | --- | --- |
| 1 | Definition and purpose | Not mentioned/incorrect | Mentioned but vague or partly correct | Clearly and correctly explained |
| 2 | Indications | Not mentioned/incorrect | Partial or nonspecific | Appropriate indications clearly stated |
| 3 | Contraindications/limitations | Not mentioned | Brief or incomplete | Clearly acknowledges limitations |
| 4 | Preoperative evaluation | Not mentioned | Brief mention only | Appropriate diagnostic evaluation described |
| 5 | Procedure | Not mentioned/incorrect | General or oversimplified | Broadly correct procedural explanation |
| 6 | Tooth preservation | Incorrect or absent | Implied only | Clearly presented as tooth-preserving |
| 7 | Outcomes/prognosis | Exaggerated/incorrect | General positive statement only | Balanced and realistic |
| 8 | Risks/complications | Not mentioned/incorrect | Brief/incomplete | Appropriate risks described |
| 9 | Postoperative care | Not mentioned/incorrect | Brief mention | Realistic recovery/care explained |
| 10 | Alternatives | Not mentioned | Vague mention | Alternatives appropriately acknowledged |
| 11 | Misleading simplification | Major distortion | Some oversimplification | Balanced presentation |
| 12 | Overall consistency | Multiple inaccuracies | Mixed accuracy | Broadly consistent with accepted principles |

**Scoring method**

**Each item was scored on a 3-point scale: 0 = not mentioned, clearly incorrect, or seriously misleading; 1 = mentioned but incomplete, vague, oversimplified, or only partly correct; 2 = clearly stated, clinically accurate, and sufficiently explained for a patient-oriented context.**

**Total score**

**The checklist includes 12 items, giving a total score range of 0 to 24, with higher scores indicating greater clinical accuracy.**

**Interpretation of total score**

**0–8 points: low clinical accuracy**

**9–16 points: moderate clinical accuracy**

**17–24 points: high clinical accuracy**

**Evaluation approach**

**Each video was scored independently by 2 reviewers after full viewing. Scoring was based on the information actually presented in the video, including spoken explanation, on-screen text, captions, and clinically relevant visual demonstration when applicable. When disagreement occurred, consensus was reached through discussion, and a third reviewer was consulted when necessary.**

**Principles for scoring**

**A score of 2 required not only factual correctness but also sufficient clarity for a patient-oriented context. Statements that were technically correct but too brief to support reasonable patient understanding were scored 1. If both correct and incorrect statements appeared within the same domain, the score was assigned according to the likely overall effect on patient understanding. Videos were not expected to provide specialist-level technical detail; the emphasis was on clinical correctness, balance, and the avoidance of misleading simplification.**
