# Supplementary material for: Platform-mediated patient access to apical surgery information on Chinese short-video platforms: a cross-sectional study of clinical accuracy, transparency, and misinformation risk
Source: Front Oral Health. 2026 Jun 29;7:1870413. doi: 10.3389/froh.2026.1870413 (PMC13357420; doi:10.3389/froh.2026.1870413)
Supplement: Supplementary file 2 [file Table2.docx]

**Supplementary Table S2. Inter-rater agreement in the pilot calibration phase**

| Measure | Scale type | Reliability statistic | Value | Interpretation |
| --- | --- | --- | --- | --- |
| PEMAT understandability | Continuous summary score | ICC (two-way random, absolute agreement) | 0.89 | Good |
| PEMAT actionability | Continuous summary score | ICC (two-way random, absolute agreement) | 0.86 | Good |
| Clinical accuracy total score (0–24) | Continuous summary score | ICC (two-way random, absolute agreement) | 0.91 | Excellent |
| JAMA authorship | Binary categorical | Cohen’s kappa | 0.84 | Substantial |
| JAMA attribution | Binary categorical | Cohen’s kappa | 0.78 | Substantial |
| JAMA disclosure | Binary categorical | Cohen’s kappa | 0.76 | Substantial |
| JAMA currency | Binary categorical | Cohen’s kappa | 0.80 | Substantial |
| Misinformation severity (mild/moderate/severe) | Ordinal categorical | Weighted kappa | 0.82 | Substantial |
| Misleading content pattern classification | Binary categorical | Cohen’s kappa | 0.79 | Substantial |

*ICC = intraclass correlation coefficient. Inter-rater reliability was assessed in the 30-video pilot calibration set used before formal evaluation. ICCs were calculated using a two-way random-effects model with absolute agreement for continuous summary scores. Cohen’s kappa was used for binary categorical variables, and weighted kappa for ordinal categorical variables. Interpretation followed conventional thresholds, with values above 0.75 considered indicative of good agreement.*
